# Immunopathogenesis, molecular phenotyping, and host-directed interventions for severe pediatric *Mycoplasma pneumoniae* pneumonia in the post-pandemic era

**DOI:** 10.3389/fcimb.2026.1833802

**Published:** 2026-07-16

**Authors:** Fu Zhang

**Affiliations:** Department of Pediatrics, Guangyuan Central Hospital, Guangyuan, Sichuan, China

**Keywords:** gut-lung axis, host-directed therapies, immunopathogenesis, machine learning, macrolide resistance, multi-omics, severe mycoplasma pneumoniae pneumonia

## Abstract

Severe *Mycoplasma pneumoniae* pneumonia (SMPP) presents a major clinical challenge, increasingly complicated by macrolide-resistant strains (MRMP) and viral co-infections. The progression of SMPP is fundamentally driven by intricate immune-mediated mechanisms rather than direct pathogen-induced injury. Key drivers include innate immune hyperactivation, such as NLRP3 inflammasome-mediated pyroptosis, and adaptive immune dysregulation, characterized by the hyperactivation of pathogenic Th17 cells. Furthermore, systemic azithromycin administration can disrupt the gut-lung axis by depleting short-chain fatty acid (SCFA)-producing commensals, impairing regulatory T cell (Treg) generation, and exacerbating lung injury. Traditional diagnostics often struggle to predict severe disease; however, integrating multi-omics data and clinical biomarkers into machine-learning nomograms offers a more precise approach for identifying high-risk patients early. This precision risk stratification guides the deployment of targeted host-directed therapies (HDTs), including corticosteroids, small molecule inhibitors, anticoagulants, and early bronchoalveolar lavage. These interventions aim to dampen excessive inflammation, counteract hypercoagulability, and restore airway patency. This review summarizes the current understanding of SMPP and proposes a translational framework combining advanced diagnostics with targeted interventions to optimize clinical outcomes.

## 1 Introduction

### 1.1 Epidemiological evolution and economic burden

*Mycoplasma pneumoniae* is a major bacterial cause of community-acquired lower respiratory tract infections in children. Globally, it is responsible for 10% to 40% of all pediatric community-acquired pneumonia (CAP) cases (Wang et al., 2024d). As an atypical bacterium lacking a peptidoglycan cell wall, *M. pneumoniae* primarily establishes infection by attaching to the respiratory ciliated epithelium using specialized organelles, mainly P1 and P30 adhesins. This firm attachment causes direct cell damage by releasing hydrogen peroxide and Community-Acquired Respiratory Distress Syndrome (CARDS) toxin. Consequently, this localized damage triggers a strong and often excessive host inflammatory responses (Wang et al., 2024d; Zhang et al., 2024d).

After the easing of COVID-19 non-pharmaceutical interventions (NPIs), *Mycoplasma pneumoniae* pneumonia (MPP) cases have surged globally, with a particularly intense outbreak in East Asia. Because children had less exposure to typical pathogens during the pandemic, their waning herd immunity—often termed an “immunity debt”—made them highly vulnerable to widespread *M. pneumoniae* infections (Bi et al., 2025; Li et al., 2025d). Recentdata from 2023–2024 show that hospitalization rates for MPP have risen dramatically, significantly surpassing pre-pandemic levels (, Lee et al., 2024; Xu et al., 2024c; Huang et al., 2026).This shift is not limited to Asia. European cohorts, such as those in Romania, have also reported delayed but significant outbreaks, often presenting with extensive alveolar infiltrates (Ulmeanu et al., 2025). This surge has created a heavy economic burden. Over the past decade, hospitalization costs have escalated significantly, especially for severe cases that require long hospital stays and advanced immune treatments. This highlights the urgent need for better medical reimbursement policies (Shen et al., 2026).

### 1.2 Macrolide-resistance mutations and the MRMP crisis

Alongside this rise in cases,

macrolide-resistant *M. pneumoniae* (MRMP) has become a growing crisis. Strains carrying point mutations in domain V of the 23S rRNA gene (mainly A2063G and A2064G) are highly resistant to first-line macrolide therapies. These mutations alter the drug’s binding site on the bacterial ribosome, rendering conventional antibioticslike azithromycin ineffective (Zhan et al., 2022; Cheng et al., 2024; Bolormaa et al., 2025). Recent surveillance shows that MRMP prevalence now exceeds 80-90% in several pediatric cohorts in China and other Asian countries. This is largely driven by the spread of P1-type 1 (P1-I) and Sequence Type 3 (ST-3) lineages (Zhan et al., 2022; Zhang et al., 2023; Yang et al., 2024). Recent large-scale studies show that this high level of resistance leads to poor clinical outcomes. Children with MRMP often experience longer periods of fever, extended hospital stays, and a much higher risk of developing refractory *Mycoplasma pneumoniae* pneumonia (RMPP) (Desai et al., 2021; Lu et al., 2024b; Song et al., 2024).

### 1.3 The microbiological and clinical challenge of disease exacerbation

The clinical presentation of MPP has changed significantly. Historically, it was seen as a mild, self-limiting “walking” pneumonia. Today, however, we are seeing a sharp increase in severe MPP (SMPP) and RMPP. These severe cases often lead to serious complications inside and outside the lungs, including necrotizing pneumonia (NP), massive pleural effusion, plastic bronchitis (PB), acute respiratory distress syndrome (ARDS), and pulmonary thromboembolism (PTE) (Mu et al., 2024; Wang et al., 2024b; Zhang et al., 2024b; Hou et al., 2025; Tang et al., 2025).

Diagnosing these severe cases early is a major challenge. Traditionally, clinicians relied on *M. pneumoniae* -specific IgM testing. However, this method often misses early infections because antibodies take time to develop. Recent evidence shows that combining simultaneous amplification and testing (SAT) with IgM screening works much better. This combined approach accurately predicts severe disease earlier, giving doctors crucial time to intervene (Tuo et al., 2023). Furthermore, atypical presentations, such as MPP without a fever, are increasingly common. Even without a fever, these patients often suffer from severe organ impairment and small airway dysfunction, leading to delayed diagnosis and sudden deterioration (Li et al., 2024).

Systemic spread of the bacteria further complicates the disease. Emerging evidence has detected *M. pneumoniae* DNA in the peripheral blood and plasma of some pediatric patients, suggesting that *M. pneumoniae* septicemia is not uncommon and may cause distant organ damage (Liu et al., 2021). Faced with these severe and often covert phenotypes, the traditional “antibiotic-centric” diagnostic and therapeutic framework is increasingly inadequate Because these severe cases can be hard to detect and treat, the traditional approach that relies solely on antibiotics is no longer sufficient (Lloyd and Wolf, 2025; Respiratory, 2025).

A critical question remains: does the A2063G make*M. pneumoniae* naturally more virulent? Current evidence suggests that these resistance mutations do not directly increase the secretion of toxins. Instead, they allow the bacteria to survive longer and form biofilms despite antibiotic treatment. This prolonged infection constantly triggers the host’s immune system, eventually leading to hyper-inflammation (Kuo et al., 2022; Zhan et al., 2022). In many cases, patients with macrolide-sensitive strains also develop severe disease (Zhang et al., 2021). Moreover, severe lung damage (such as post-infectious bronchiolitis obliterans) often persists even after the bacteria are fully eradicated (Tsai et al., 2021; Song and Xu, 2023). This strongly suggests that SMPP is driven more by the host’s exaggerated immune response than by the bacteria alone.

## 2 Core pathogenesis: pathogen-host interactions and immunopathology

**Figure 1:**
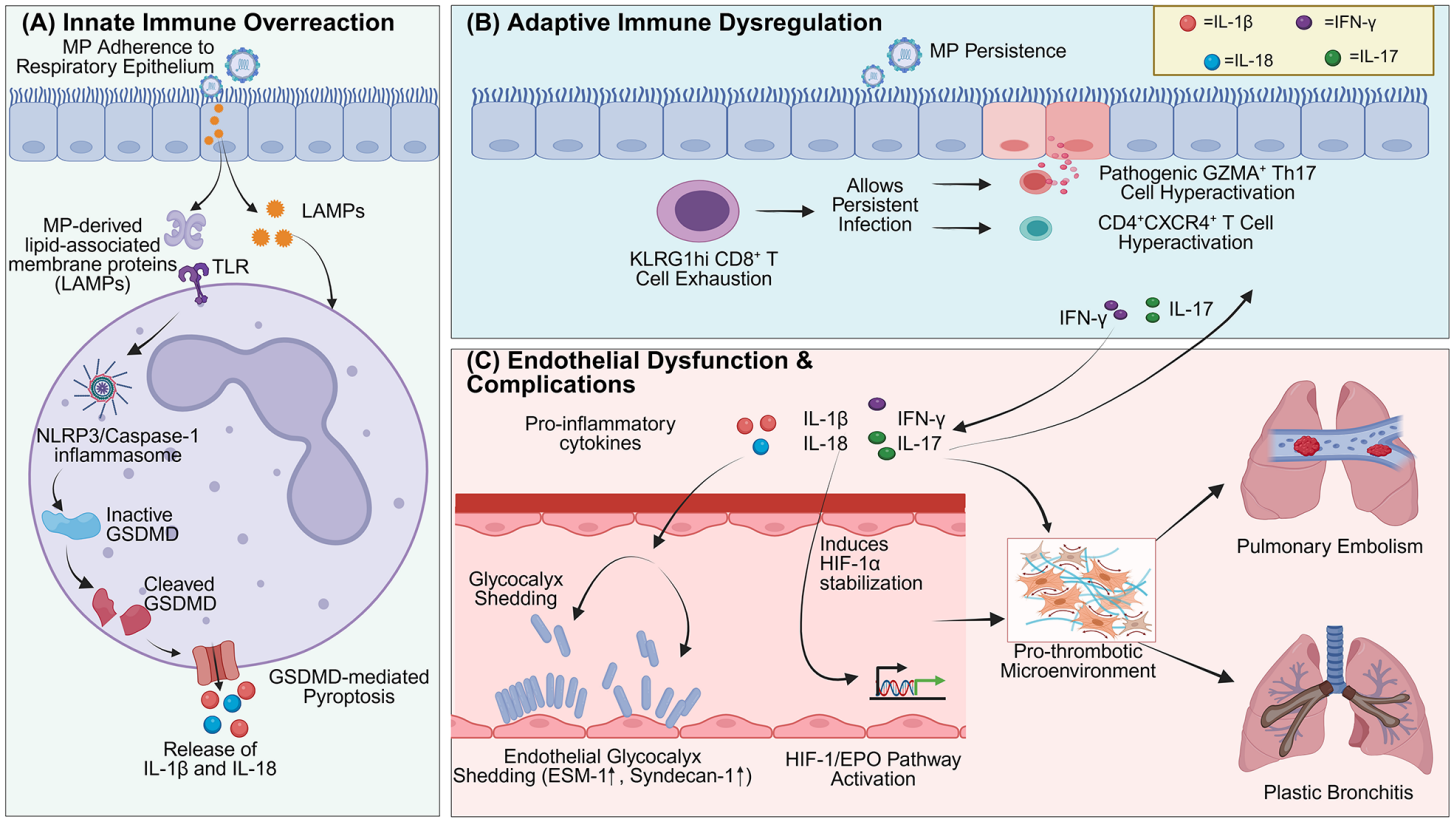
Immunopathogenesis of Severe *Mycoplasma pneumoniae* Pneumonia (SMPP). The fundamental progression of SMPP is driven by complex host immune responses. Symbols: Red circles denote IL-1β, blue circles denote IL-18, purple circles denote IFN-γ, and green circles denote IL-17. The orange cogwheels represent LAMPs, and the horseshoe structures represent TLRs. **(A)** Innate immune overreaction triggered by *M. pneumoniae* adherence. *M. pneumoniae* -derived lipid-associated membrane proteins (LAMPs) activate the NLRP3/Caspase-1 inflammasome. This leads to the cleavage of GSDMD (the blue form represents the inactive precursor, while the red form represents the active cleaved form), which forms membrane pores resulting in pyroptosis and the massive release of pro-inflammatory cytokines (IL-1β and IL-18). **(B)** Adaptive immune dysregulation. Persistent *M. pneumoniae* infection induces KLRG1hi CD8+ T cell exhaustion and promotes the hyperactivation of pathogenic GZMA+ Th17 cells and CD4+CXCR4+ T cells, resulting in excessive IFN-γ and IL-17 production. **(C)** Endothelial dysfunction and vascular complications. The cytokine storm induces endothelial glycocalyx shedding (upregulation of ESM-1 and Syndecan-1) and activates the HIF-1/EPO pathway, creating a pro-thrombotic microenvironment (depicted by RBC clusters and fibrin networks) that contributes to severe complications such as pulmonary embolism and plastic bronchitis.

The pathogenesis of SMPP reflects a classic model of infection-induced, immune-mediated injury. Localized epithelial damage from *M. pneumoniae* adhesion rapidly escalates into an uncontrolled systemic inflammatory storm. This process involves three key components: adaptive immune deviation, innate immune hyperactivation, and profound endothelial dysfunction (**Figure 1**) (Lai et al., 2022; Yu et al., 2024; Jia et al., 2025a).

### 2.1 Adaptive immune dysregulation and cellular heterogeneity

The polarization and functional status of T and B lymphocytes critically dictate the trajectory of *M. pneumoniae* infection. Recent advancements utilizing single-cell RNA sequencing (scRNA-seq), high-dimensional flow cytometry, and Cell Population Data (CPD) have unveiled a distinct immunological landscape in children with SMPP (Zhan et al., 2025; Zhang et al., 2025b; Li et al., 2026a).

During acute severe infection, peripheral T lymphocyte counts often drop, particularly the CD3+ and CD4+ subsets. This suggests immune exhaustion or tissue sequestration (Zhan et al., 2025). SMPP is uniquely characterized by a significant expansion of effector CD8+ T cells alongside a paradoxical prevalence of exhausted KLRG1hi CD8+ T cell compartments. This exhaustion phenotype compromises effective bacterial clearance and promotes persistent colonization within the respiratory tract (Jia et al., 2025a; Zhang et al., 2025b). Concurrently, there is an overactivation of pathogenic GZMA+ (Granzyme A-producing) Th17 cells and CD4+CXCR4+ T cells, which are directly correlated with disease severity, lung consolidation, and prolonged fever (Jia et al., 2025a; Li et al., 2025a). The enhanced expression of the Notch ligand DLL4 on peripheral blood mononuclear cells actively promotes Th1/Th17-mediated immune responses while suppressing protective Th2 pathways. This cellular shift creates an aggressive pro-inflammatory milieu dominated by significantly elevated levels of IFN-γ, TNF-α, IL-17, IL-2, and IL-6 in both serum and bronchoalveolar lavage fluid (BALF). Notably, the concentration of cytokines such as TNF-α and IL-6 demonstrates a strong inverse correlation with the percentage of normal lung density on CT scans, underscoring their role in parenchymal destruction (Dong et al., 2025a; Wang et al., 2025b).

In SMPP, IgM+ plasma cells and unswitched memory B cells are hyperactivated, raising the proportion of CD3-CD19+ B cells. This elevation, together with increased monocyte counts, is an independent predictor of severe disease (Han et al., 2024; Zhan et al., 2025). This dysregulated B-cell response accounts for the robust IgM seroconversion and the generation of aberrant autoantibodies. These autoantibodies frequently cross-react with host tissues—such as anticardiolipin antibodies and anti-nuclear antibodies—exacerbating systemic injury and substantially contributing to hypercoagulable states and immune complex deposition (Jiang et al., 2024; Zhou et al., 2024).

### 2.2 Innate immune hyperactivation, oxidative stress, and pyroptosis

The innate immune system, specifically macrophages and neutrophils, acts as the primary amplifier of the inflammatory cascade in SMPP. *M. pneumoniae* interacts with innate immune cells mainly through lipid-associated membrane proteins that trigger Toll-like receptors (TLRs), leading to oxidative stress and inflammasome activation. Clinical studies highlight the significant elevation of oxidative stress markers, such as malondialdehyde (MDA) and advanced oxidation protein products (AOPP), alongside a concerning depletion of protective superoxide dismutase (SOD) in the BALF of children with RMPP (Wang et al., 2024c). Interestingly, the baseline immune competence heavily influences this innate response; retrospective case-control studies have identified that children with MPP exhibit significantly lower peripheral blood levels of essential vitamins (Vitamin A, D, B1, B7, and C). These vitamin deficiencies correlate negatively with neutrophil percentages and independently predict higher susceptibility to severe *M. pneumoniae* infection (Li et al., 2026a).

Furthermore, molecular studies have elucidated the critical role of ATP-binding cassette transporter G1 (ABCG1) in MPP. *M. pneumoniae* infection downregulates ABCG1 in neutrophils, triggering NLRP3/Caspase-1 inflammasome activation. This pathway induces Gasdermin D (GSDMD)-dependent neutrophil pyroptosis, resulting in the massive extracellular release of highly pyrogenic cytokines (IL-1β and IL-18), thereby fueling the “cytokine storm” and massive airway inflammation (Cai et al., 2025). Novel innate immune markers such as soluble triggering receptor expressed on myeloid cells-1 (sTREM-1) and interferon-inducible protein-10 (IP-10/CXCL10) are profoundly elevated in SMPP. Elevated CXCL10 act as a chemoattractant, drawing hyperactive innate cells and CXCR3+ Th1 cells into the lung parenchyma—a process linked to disease severity and tissue damage (Bai et al., 2021; Li et al., 2021; Xu et al., 2024a).

Macrophage-epithelial crosstalk is fundamentally altered. Concurrently, IL-17 stimulation prompts neutrophils to release damage-associated molecular patterns (DAMPs), notably the calprotectin complex S100A8/A9. In the BALF of SMPP patients, highly elevated S100A8/A9 directly binds to local alveolar epithelial cells, triggering severe epithelial apoptosis and contributing to the catastrophic loss of alveolar integrity observed in necrotizing pneumonia. Clinical utility studies confirm that combining serum S100A8/A9 with CRP yields an exceptional predictive value (AUC >0.90) for assessing MPP severity (Fu et al., 2022; Chen et al., 2023). The release of autotaxin, an autocrine motility factor, worsens structural lung damage, correlating with elevated IL-6 and IL-8 levels (Guan et al., 2025). Systemic inflammation often spills over to extra-pulmonary organs, causing myocardial injury (linked to altered neutrophil-to-lymphocyte ratio[NLR]) and early renal tubular damage (shown by raised urinary N-acetyl-β-D-glucosaminidase [NAG] and α1-microglobulin) (Gu et al., 2024; Ni and Zhao, 2025).

### 2.3 Coagulation-inflammation cascade and endothelial injury

A defining characteristic of modern SMPP is the high incidence of hypercoagulability, manifesting as plastic bronchitis (characterized by fibrin-rich bronchial casts) and pulmonary thromboembolism (Zhang et al., 2024a; Wang et al., 2025a; Yu et al., 2025). This hypercoagulable state is deeply intertwined with immune-mediated endothelial damage.

Elevated pro-inflammatory cytokines activate the Hypoxia-Inducible Factor-1 (HIF-1)/Erythropoietin (EPO) pathway. This raises whole blood viscosity, erythrocyte aggregation index, RBC counts, and hematocrit—changes that promote localized hypoxia and micro-thrombosis (Zheng et al., 2021). Moreover, the shedding of Syndecan-1 and elevated Endothelial cell-specific molecule 1 (ESM-1) indicate severe endothelial glycocalyx degradation (Li et al., 2023b). This shedding exposes pro-thrombotic sub-endothelial matrix proteins to the bloodstream. Endothelial injury, intense inflammation, and coagulation (shown by elevated D-dimer, fibrinogen, and fibrin degradation products [FDP]) together drive the development of airway necrosis and systemic embolism in children. In clinical practice, a D-dimer level surpassing 3.705 mg/L or 2.44 mg/L has been repeatedly validated as an independent and strong predictor for the composite outcome of necrotizing pneumonia and plastic bronchitis (Li et al., 2023b; Dong et al., 2025b; Korneenko et al., 2025; Yue et al., 2025).

## 3 Co-infections and alterations in respiratory and gut microbiota

### 3.1 Metagenomic profiling and radiological features of co-infections

The lifting of strict NPIs has led to a dramatic surge in respiratory co-infections, heavily complicating the clinical course of MPP. Targeted next-generation sequencing (tNGS) and multiplex PCR studies reveal that approximately 40% to 50% of severe MPP cases harbor co-detectable respiratory pathogens (Wang et al., 2021; Chi et al., 2024; Fu et al., 2024);. Common viral co-pathogens include human mastadenovirus (HAdV), rhinovirus, respiratory syncytial virus, and influenza A virus (Wang et al., 2021; Fu et al., 2024; Piao et al., 2025). Bacterial co-infections are predominantly driven by Streptococcus pneumoniae, Haemophilus influenzae, and Chlamydia pneumoniae. Interestingly, comparative analyses show that Chlamydia pneumoniae co-infections tend to occur in slightly older children and present with more prominent chest pain and notably higher eosinophil counts compared to pure MPP (Wang et al., 2021; Lv et al., 2024; Huang et al., 2025). Furthermore, in certain endemic regions, concurrent Mycobacterium tuberculosis infections causing tracheobronchial tuberculosis alongside MPP represent a severe diagnostic challenge, heavily relying on advanced machine learning algorithms (like XGBoost) for accurate differentiation (Zhou et al., 2022b).

To rapidly differentiate viral pneumonia from MP mono-infection prior to pathogen sequencing, researchers have successfully developed cytokine-based nomograms; models incorporating the TNF-α/IL-10 ratio, IL-8, and procalcitonin exhibit excellent discriminatory power (AUC = 0.878) (Deng et al., 2023). The presence of these co-pathogens exerts a synergistic amplification on disease severity. For instance, co-infection with HAdV or influenza A virus significantly elevates systemic inflammatory markers (e.g.,IL-6, LDH, D-dimer) and the compensatory anti-inflammatory cytokine IL-10, prolongs the duration of fever, and increases the likelihood of progressing to lobar pneumonia compared to *M. pneumoniae* mono-infection (Zhou et al., 2022b; Lv et al., 2024; Huang et al., 2025; Piao et al., 2025). Mixed infections also present distinct radiological challenges; comparative chest CT analyses indicate that while pure MPP often manifests as interstitial changes, co-infections with Streptococcus pneumoniae are characterized by a markedly higher incidence of ground-glass opacities, thickened vascular bundles, substantial pleural effusion volumes, and larger transverse diameters of enlarged mediastinal lymph nodes (Liu et al., 2024).

### 3.2 Dysbiosis of the respiratory microbiota and the “gut-lung axis”

Beyond specific pathogens, the overall ecological balance of the lower respiratory tract (LRT) microbiota is severely disrupted during RMPP. Metagenomic analyses of BALF demonstrate that while the phylum *Tenericutes* dominates mono-infections, co-infections with viruses such as HAdV shift the dominant microbiota composition significantly, increasing the relative abundance of *Preplasmiviricota* and other opportunistic taxa like *Trichoderma citrinoviride* (Zhou et al., 2022b; Xi et al., 2024).

**Figure 2:**
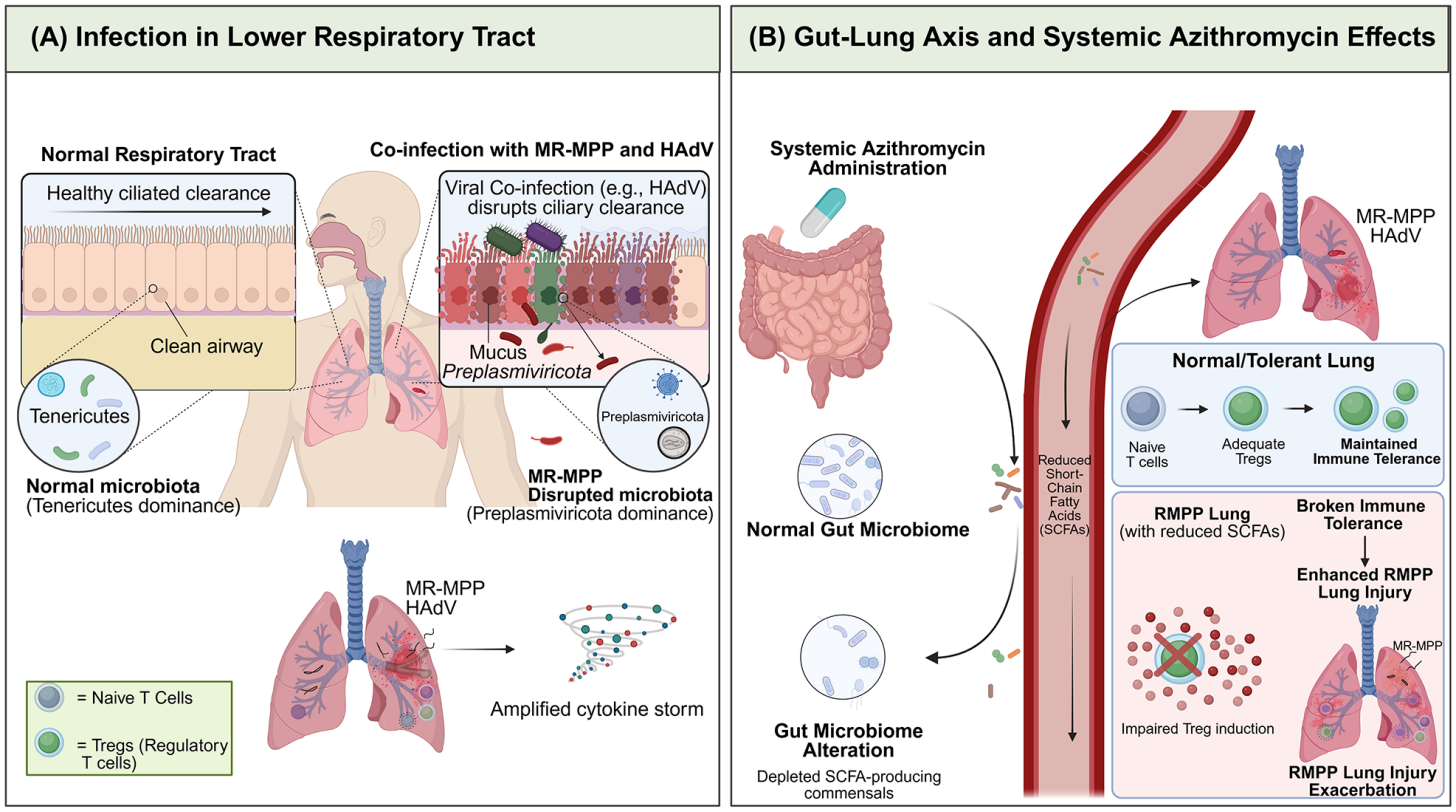
Impact of viral co-infections and macrolide-induced gut-lung axis dysbiosis in SMPP. Symbols: Blue circles denote Naive T cells; green circles denote Tregs (Regulatory T cells); specific icons denote bacteria (rod-shaped) and viruses (icosahedral virions for Preplasmiviricota). **(A)** Infection microenvironment in the lower respiratory tract. Co-infection with macrolide-resistant *M. pneumoniae* (MR-MPP) and human adenovirus (HAdV) disrupts healthy ciliated clearance and alters the normal respiratory microbiota (shifting from *Tenericutes* to *Preplasmiviricota* dominance). This synergistic disruption amplifies the cytokine storm (depicted by a cluster of multi-colored cytokine dots). **(B)** Disruption of the gut-lung axis by systemic azithromycin effects. Systemic administration of azithromycin alters the normal gut microbiome, depleting short-chain fatty acid (SCFA)-producing commensals. The resulting reduction in circulating SCFAs impairs the generation of regulatory T cells (Tregs) in the lungs (indicated by a red cross over the Treg). This breakdown of immune tolerance (indicated by a standard downward causal arrow) ultimately exacerbates RMPP-induced lung injury.

Crucially, the “Gut-Lung Axis” represents a vital, yet underappreciated, bidirectional communication network in MPP. Prolonged macrolide (azithromycin) therapy significantly perturbs the intestinal flora. 16S rRNA gene sequencing reveals that azithromycin administration in MPP children induces an abnormal enrichment of *Haemophilus* and *Pasteurellales* in the gut, alongside a depletion of beneficial commensals (Deng et al., 2023). According to established immunological principles, such gut dysbiosis inherently reduces the production of microbiota-derived metabolites, such as short-chain fatty acids (SCFAs). SCFAs play a key role in maintaining regulatory T cells (Tregs). The depletion of these metabolites disrupts Th17/Treg immune tolerance in the lungs, creating a pro-inflammatory feedback loop that exacerbates airway injury (**Figure 2**) (Deng et al., 2023).

## 4 Multi-omics phenotyping and host-response biomarker signatures

### 4.1 Traditional serum, imaging, and BALF biomarker models

Recognizing the limitations of relying solely on microbiological diagnostics and delayed seroconversion, researchers have increasingly turned to early host-response biomarkers. Advanced machine learning algorithms—including XGBoost, LightGBM, and CatBoost—have been developed to transform routine clinical variables into highly precise predictive tools. For instance, models utilizing the CatBoost algorithm, augmented by Shapley Additive Explanations (SHAP) for transparent clinical interpretation, have identified fever duration, D-dimer, platelets (PLT), C-reactive protein (CRP), lactate dehydrogenase (LDH), and NLR as the top features for predicting SMPP, achieving remarkable accuracy (Gong et al., 2021; Zhang et al., 2024c; Li et al., 2025c; Liu et al., 2025; Mu et al., 2025b; Ye et al., 2025). Recently, web-based dynamic online nomograms have been introduced to provide real-time risk stratification at the bedside (Jian et al., 2025).

**Table 1** summarizes the emerging predictive biomarkers and multi-omics signatures utilized in recent clinical models. Key biological markers consistently identified as independent predictors of SMPP, NP, and PB include LDH, D-dimer, CRP, PCT, and inflammatory indices like the NLR and Systemic Immune-inflammation Index (SII) (Huang et al., 2021b; Jia et al., 2024; Papan et al., 2024; Xiong et al., 2024; He et al., 2025; Jia et al., 2025b). Specific cut-off values—such as LDH > 378–400 U/L, D-dimer > 1.5-2.4 mg/L, and CRP > 40 mg/L—are strongly indicative of progression to plastic bronchitis or pulmonary embolism (Desai et al., 2021; Li et al., 2022).

**Table 1:** Key host-response biomarkers and multi-omics signatures for predicting severe/refractory MPP.

Biomarker category	Specific markers	Primary biological/pathological role	Clinical predictive value	Validation stage/evidence level	Ref.
Routine Biochemical & Coagulation	LDH, Ferritin, CRP	Markers of intense systemic inflammation and cell necrosis.	Nomogram integration yields AUC >0.90 for SMPP/RMPP. Cut-offs: LDH >400 U/L, CRP >40 mg/L.	Retrospective & Prospective Clinical Cohorts	(Jia et al., 2024; Papan et al., 2024; Xiong et al., 2024; He et al., 2025)
	D-dimer, Fibrinogen, FDP	Reflects hypercoagulable state and endothelial damage.	Strong independent predictor for pulmonary embolism and plastic bronchitis (D-dimer >2.44 mg/L).	Large-scale Retrospective Cohorts	(Desai et al., 2021; Li et al., 2022; Korneenko et al., 2025)
Cellular Immune Ratios & Morphologies	NLR, SII, CD3-CD19+ B cell ratio, CPD	Indicates innate immune hyperactivation and leukocyte morphological shifts.	High sensitivity for differentiating RMPP from general MPP; incorporated in CatBoost models.	Machine Learning Validated Cohorts	(Huang et al., 2021b; Yu et al., 2024; Li et al., 2025c)
Cytokines & Chemokines	IL-6, IL-17, IP-10 (CXCL10), sTREM-1	Mediates “cytokine storm”, neutrophil chemotaxis, and alveolar apoptosis.	Serum/BALF levels correlate directly with the extent of lung consolidation and necrosis.	Case-Control/Prospective Cohorts	(Bai et al., 2021; Li et al., 2021; Xu et al., 2024a)
Imaging Biomarkers	LUS Score, Qanadli Score	Quantifies pulmonary consolidation, pleural effusion, and arterial occlusion.	LUS >8 predicts SMPP; high Qanadli score predicts severity of PE.	Prospective Validation	(Dai et al., 2021, 2026)
Airway Remodeling	YKL-40, MUC5AC	Associated with mucus hypersecretion and structural airway damage.	Predicts the necessity and timing for interventional bronchoscopy (BAL).	Retrospective Cohorts	(Huang et al., 2021a; Mu et al., 2025a)
Non-coding RNAs (ceRNA)	*circ_0054633*, *miR-29c*, *miR-146a*, *miR-1323*	Acts as miRNA sponges; regulates post-transcriptional expression of IL-6 and IL-17 pathways.	Highly sensitive molecular phenotypes differentiating RMPP at the transcriptomic level.	*In vitro* & Clinical Validation	(Wei et al., 2024; Shen et al., 2025; Wang et al., 2025b, 2025; Li et al., 2026b)
Metabolomics & Urinary Markers	Acetylphosphate, 2,5-dioxopentanoate, Urinary NAG	Reflects disrupted energy metabolism and early renal tubular injury due to immune complexes.	Potential non-invasive urinary diagnostic/complication biomarkers for MPP.	Discovery Cohort/LC-MS/MS	(He et al., 2023; Ni and Zhao, 2025)

Emerging imaging and cellular biomarkers also enhance predictive accuracy. The Lung Ultrasound (LUS) score has been validated as a robust, non-ionizing, bedside predictor; patients with LUS scores >8 combined with prolonged fever and elevated PCT are at significantly heightened risk for SMPP (Dai et al., 2026). Moreover, the integration of CPD derived from routine hematology analyzers (such as LY-X and NE-WY indices) provides real-time morphological changes in peripheral leukocytes, serving as early surrogate markers for disease progression (Wang et al., 2023a).

Additionally, the combinatorial BV score (incorporating TRAIL, IP-10, and CRP) has demonstrated superior discriminatory accuracy for distinguishing inflammation induced by *M. pneumoniae* from typical viral or pyogenic bacterial etiologies (Wang et al., 2024e). Markers of airway remodeling and mucus hypersecretion are also gaining attention. Significantly elevated levels of Chitinase-like protein YKL-40 and Mucin 5AC (MUC5AC) in sputum or BALF strongly predict refractory disease courses and the necessity for interventional bronchoscopy (Huang et al., 2021a; Mu et al., 2025a).

### 4.2 Emerging transcriptomic and ceRNA networks

Multi-omics approaches offer a deeper resolution of the molecular phenotyping of MPP. Urine metabolomic profiling via LC-MS/MS has successfully identified metabolites such as acetylphosphate and 2,5-dioxopentanoate as potential non-invasive diagnostic biomarkers, shedding light on the disrupted energy and amino acid metabolism in affected children (He et al., 2023).

More importantly, the non-coding RNA network—particularly the competing endogenous RNA (ceRNA) mechanism—has emerged as a central epigenetic regulator of the MPP immune response. Circular RNAs (circRNAs) and long non-coding RNAs (lncRNAs) act as miRNA sponges: they bind specific miRNAs and prevent them from repressing pro-inflammatory mRNAs. For instance, the drastic upregulation of *circ_0054633* serves as a robust diagnostic biomarker for RMPP (Shen et al., 2025). Mechanistically, whole-blood RNA-sequencing reveals that specific circRNA-mRNA networks are highly enriched in the IL-17 signaling pathway, actively driving the hyper-inflammatory state in children with RMPP (Wang et al., 2025c). Downregulated of *miR-1323*, for example, lifts post-transcriptional repression of *IL6*, promoting cytokine overproduction in macrophages (Yin et al., 2021). Similarly, decreased expression of *miR-29c* and *miR-146a* correlates strongly with disease severity and intense inflammatory mediator release (Wei et al., 2024; Li et al., 2026b).

## 5 HDTs and precision interventions

### 5.1 Overview of available antibiotic treatment options and their limitations

Traditional macrolide antibiotics, primarily azithromycin and clarithromycin, have long served as the cornerstone of empirical therapy for MPP due to their favorable safety profile in pediatrics and excellent intracellular penetration. By targeting the bacterial ribosome, these agents effectively inhibit protein synthesis (Krawczyk et al., 2024). However, the clinical utility of this first-line approach is increasingly constrained by two major limitations: adverse gastrointestinal effects, such as antibiotic-associated diarrhea (AAD) (Ling et al., 2018), and the alarming global escalation of MRMP (Yin et al., 2017).

When macrolides fail or MRMP is suspected, alternative antimicrobial agents, such as tetracyclines and fluoroquinolones, demonstrate high efficacy with virtually no signs of resistance (Yin et al., 2017). Nevertheless, their application, particularly in pediatric populations, is severely limited by critical safety concerns (Yang, 2019). Tetracyclines carry classical risks of tooth discoloration and enamel hypoplasia in young children (Chinese Medical Association et al., 2026), while fluoroquinolones are associated with potential cartilage toxicity and musculoskeletal adverse events (Li et al., 2022; Yang, 2019). Consequently, the therapeutic window for these alternative options is extremely narrow in pediatric MPP, often restricting their use to older patients or refractory cases.

Furthermore, beyond direct side effects and restricted age indications, the systemic administration of these available antibiotics can profoundly disrupt the host microbiome—particularly the gut–lung axis (Song et al., 2024). Microbial dysbiosis in early life can impair immune homeostasis and is associated with negative long-term respiratory outcomes, such as the development and persistence of asthma (Valverde-Molina and Garcia-Marcos, 2023). The alteration of the gut microenvironment and bidirectional gut-lung communication further highlights the systemic consequences of antibiotic exposure.

To mitigate this dysbiosis and preserve long-term lung health, contemporary antibiotic stewardship in MPP must emphasize strict limitations on antibiotic duration to avoid unnecessary repeated courses. Additionally, incorporating adjunctive microbiome-preserving strategies, such as the co-administration of targeted probiotics (e.g., live *Clostridium butyricum* and *Bifidobacterium infantis*), represents a promising approach to partly reconstruct the gut microbiota, improve intestinal mucosal barriers, and effectively prevent AAD (Ling et al., 2018).

While navigating these available antibiotic options and their inherent clinical and ecological limitations is critical for initial management, the specific molecular mechanisms driving *M. pneumoniae* resistance and the emerging targeted strategies designed to overcome this antimicrobial resistance will be exclusively discussed in the subsequent section.

### 5.2 Antimicrobial strategies overcoming resistance

Given the high prevalence of A2063G/A2064G mutations, clinical management of severe cases frequently necessitates stepping beyond traditional macrolides, a reality underscored by rigorous placebo-controlled trials such as the MYTHIC study evaluating macrolide efficacy (Meyer Sauteur et al., 2024). Novel tetracyclines (doxycycline, minocycline) and fluoroquinolones (levofloxacin, moxifloxacin) have demonstrated superior efficacy in promoting rapid defervescence, curtailing disease progression, and reducing the length of hospital stay in MRMP pneumonia (Zhou et al., 2022a; Zhang et al., 2023; Yang et al., 2024; Zhang et al., 2025a). Notably, the early oral administration of doxycycline not only mitigates the inflammatory surge but has also been shown to reduce the overall incidence of clinical macrolide resistance by disrupting early bacterial protein synthesis before the massive cytokine storm erupts (Wang et al., 2024a). However, their pediatric application requires careful consideration of age-specific contraindications, emphasizing the need for robust susceptibility monitoring and strict risk-benefit analysis (Tao et al., 2025).

### 5.3 HDTs targeting immune overactivation and endothelial protection

**Figure 3:**
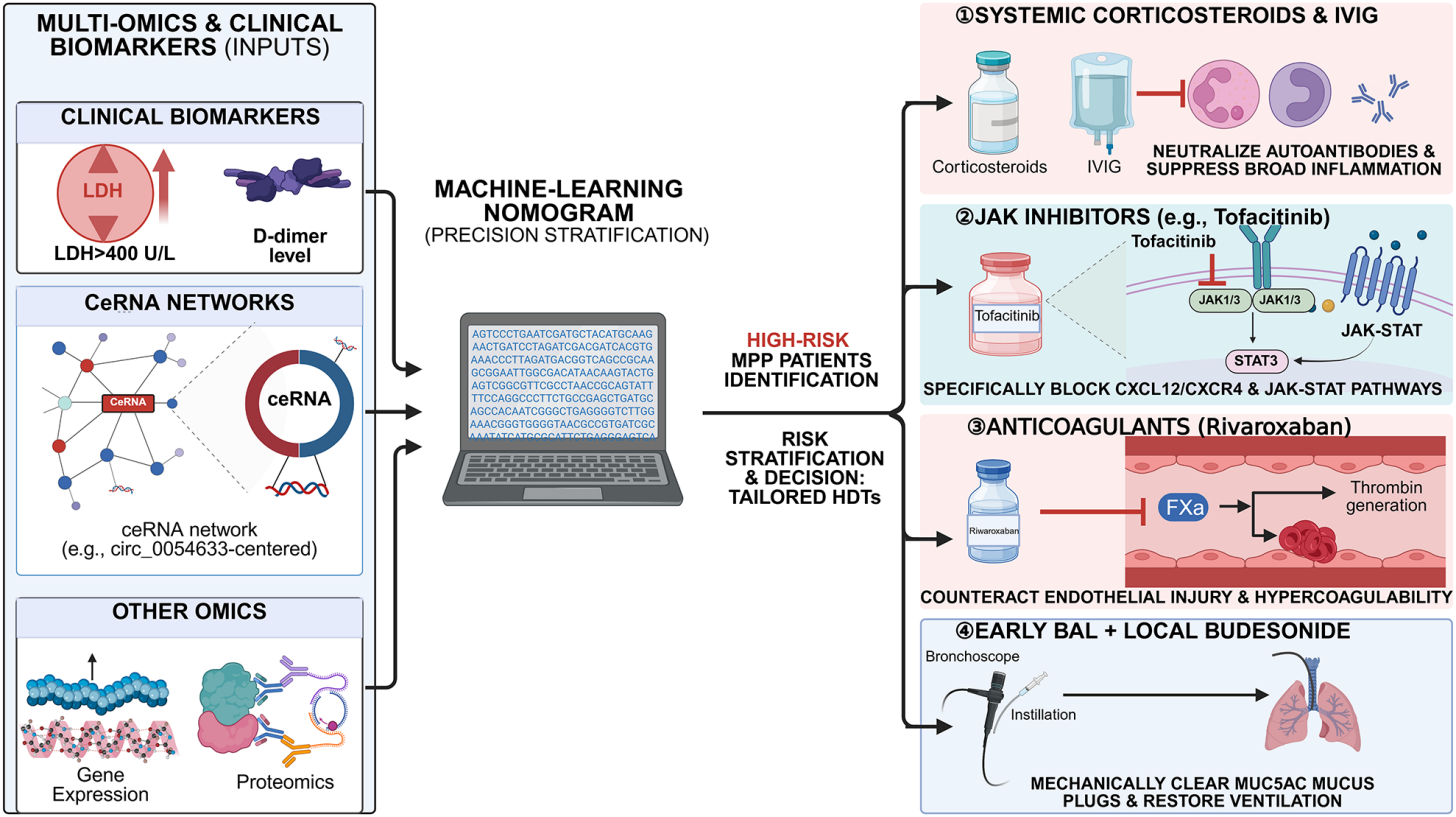
A translational framework integrating multi-omics and machine learning for precision Host-Directed Therapies (HDTs). This workflow illustrates the paradigm shift from empirical treatment to precision medicine in SMPP. Symbols: The laptop/computer icon represents machine learning algorithms processing clinical and omics data. Red lines with perpendicular bars (T-bars) represent pharmacologic inhibition. The syringe icon denotes drug instillation. Clinical biomarkers (e.g., elevated LDH > 400 U/L, D-dimer) and multi-omics inputs (ceRNA networks such as *circ_0054633*) are integrated into a machine-learning nomogram. This algorithm enables the precise identification and risk stratification of high-risk MPP patients. Based on the stratification, tailored HDTs are deployed: ① Systemic corticosteroids and IVIG to neutralize autoantibodies and suppress broad inflammation (indicated by a T-bar inhibition symbol between IVIG and autoantibodies); ② JAK inhibitors (e.g., Tofacitinib) to specifically block the CXCL12/CXCR4 and JAK-STAT pathways (with the T-bar inhibition symbol pointed directly to intracellular JAK1/3); ③ Anticoagulants (e.g., Rivaroxaban) to counteract endothelial injury and hypercoagulability by directly blocking factor Xa (FXa, indicated by a T-bar inhibition symbol pointing to FXa); ④ Early bronchoalveolar lavage (BAL) combined with local budesonide (depicted by a bronchoscope and an instillation syringe) to mechanically clear MUC5AC mucus plugs and restore airway ventilation.

The essence of managing SMPP lies in Host-Directed Therapies (HDTs) that dampen the excessive immune-inflammatory response without compromising pathogen clearance (**Table 2**; **Figure 3**).

**Table 2:** Emerging host-directed therapies (HDTs) and precision interventions in severe MPP.

Therapeutic strategy	Agent/intervention	Mechanism of action (Host-Directed)	Key clinical benefits	Validation stage/evidence level	Ref.
Broad Immunomodulation	Systemic Corticosteroids (e.g., Methylprednisolone)	Broadly suppresses inflammatory cytokine transcription; stabilizes endothelial barrier.	Rapid defervescence; prevents bronchiolitis obliterans when timed via LDH/CRP thresholds.	Clinical Guidelines & Extensive Cohorts	(Wang et al., 2023b, 2023; Wu et al., 2023; Lu et al., 2024a; Liang et al., 2026)
	Intravenous Immunoglobulin (IVIG)	Neutralizes circulating autoantibodies; provides passive immunity and dampens severe cytokine storms.	Accelerates recovery in RMPP and limits extrapulmonary complications.	Meta-analyses & RCTs	(Gao et al., 2024)
Targeted Small Molecule Inhibitors	Tofacitinib (JAK Inhibitor)	Inhibits JAK-STAT and NF-κB pathways; blocks the CXCL12/CXCR4 chemokine axis.	Profoundly reduces pro-inflammatory cytokine production and CD4+ T cell infiltration in animal models.	*In vivo* Murine Models (Preclinical)	(Li et al., 2025a)
Vascular & Endothelial Protection	Anticoagulants (Heparin, Rivaroxaban)	Reverses hypercoagulable states induced by HIF-1/EPO and endothelial shedding.	Effectively resolves cardiac and pulmonary thromboembolism; prevents necrosis.	Clinical Cohorts (Off-label consensus)	(Zhang et al., 2025a)
Airway Microenvironment Modulation	Early Bronchoalveolar Lavage (BAL)	Physically clears fibrin-rich mucus plugs (MUC5AC) and removes local inflammatory mediators.	Re-expands atelectatic lobes; drastically reduces fever duration and prevents delayed radiological resolution.	Propensity Score Matched Cohorts	(Chen et al., 2021; Leng et al., 2022; Shen et al., 2024)
	Local Instillation (Budesonide, Acetylcysteine)	Exerts direct topical anti-inflammatory and mucolytic effects at the site of consolidation.	Synergizes with BAL to improve pulmonary ventilation function and reduce systemic side effects.	Retrospective Cohorts & Clinical Trials	(Xu et al., 2024b; Li et al., 2025b)

#### 5.3.1 Targeted immunomodulation

The timely administration of systemic corticosteroids is critical for RMPP. However, the exact dosage and timing remain highly debated. Studies indicate that while high-dose methylprednisolone (> 2 mg/kg/d) facilitates faster fever resolution and enhanced radiological improvement compared to low-dose therapies, it is associated with a higher incidence of adverse gastrointestinal and dermatological reactions. High-dose therapy is typically reserved for younger children with extensive atelectasis and elevated platelet counts (Wang et al., 2023b). Timing is equally crucial; initiating glucocorticoid therapy earlier (e.g., after 3 days of persistent fever) rather than later (7 days) significantly reduces the rate of progression to severe pneumonia and shortens hospital stays (Wu et al., 2023). Guided by biomarker thresholds (e.g., LDH > 474 U/L, Ferritin elevation), timely steroid use prevents severe sequelae such as bronchiolitis obliterans (Wang et al., 2023c; Lu et al., 2024a; Liang et al., 2026). The synergy between methylprednisolone and azithromycin rapidly reduces Fractional Exhaled Nitric Oxide (FeNO) and peripheral blood eosinophils, marking the profound suppression of eosinophilic airway hyperreactivity (Zhou et al., 2022a). Additionally, intravenous immunoglobulin (IVIG) acts as a potent immunomodulator, neutralizing autoantibodies and dampening the cytokine storm, showing excellent clinical efficacy in severe, complicated cases (Gao et al., 2024). Certain Chinese herbal extracts, such as Andrographolide sulfonate, have also demonstrated potential in modulating IgG/IgA levels and ameliorating liver function concurrently during MPP treatment (Xu et al., 2024b).

#### 5.3.2 Targeted small molecule inhibitors

Experimental models have highlighted the immense potential of targeted Small Molecule Inhibitors. For example, the administration of Tofacitinib (a JAK inhibitor) in murine MPP models successfully suppressed the CXCL12/CXCR4 axis and JAK-STAT/NF-κB pathways, radically reducing pro-inflammatory cytokine production and immune cell infiltration in lung tissues (Wang et al., 2025b).

#### 5.3.3 Anticoagulation

To counter immune-mediated endothelial damage and hypercoagulability, prophylactic and therapeutic anticoagulation is increasingly utilized. Quantitative assessment of thrombus burden via the Qanadli score in CT pulmonary angiography helps guide clinical urgency (Dai et al., 2021). Low-molecular-weight heparin sequentially transitioned to oral rivaroxaban has proven highly effective and safe in resolving pulmonary and cardiac thromboembolism associated with *M. pneumoniae*, demonstrating a high rate of thrombus resolution without severe bleeding events (Zhang et al., 2025a).

#### 5.3.4 Translational barriers in pediatrics

Despite the promising potential of these HDTs (Zumla et al., 2016), significant translational barriers exist in pediatric populations. The application of targeted Small Molecule Inhibitors (e.g., Tofacitinib) and novel oral anticoagulants (e.g., Rivaroxaban) often falls under off-label use in children. Stringent ethical considerations, combined with a lack of pediatric-specific pharmacokinetic/pharmacodynamic (PK/PD) data, pose substantial hurdles (van den Anker et al., 2018). Furthermore, the risk of profound immunosuppression from JAK inhibitors or bleeding events from anticoagulants demands rigorous, biomarker-guided patient selection and cautious dose-finding in future pediatric clinical trials (Ruperto et al., 2021).

### 5.4 Airway microenvironment intervention and mitigating delayed radiographic resolution

Bronchoalveolar lavage (BAL) via flexible fiberoptic bronchoscopy serves a dual therapeutic purpose: mechanically removing fibrin-rich mucus plugs (preventing the asphyxiating complications of plastic bronchitis) and altering the local inflammatory microenvironment (Chen et al., 2021; Shen and Sun, 2024; Luo et al., 2025). The predictive evaluation of sputum MUC5AC levels, Bronchial Insufflation Sign Scores on lung ultrasound, and CT consolidation scores allows for the precise optimization of BAL timing (Huang et al., 2021a; Leng et al., 2022; Sheng et al., 2024).

Delayed radiographic resolution remains a significant clinical challenge in lobar MPP, strongly associated with elevated serum levels (e.g., LDH ≥ 378 U/L, CRP ≥ 25.92 mg/L) and prolonged hospitalization (≥ 10.5 days) (Li et al., 2023a). Propensity score matching (PSM) cohort studies indicate that early BAL intervention—specifically executed within 7 to 11.5 days of disease onset—effectively reverses this trajectory. Such timely intervention maximizes lung re-expansion, mitigates the risk of delayed radiographic resolution, and significantly curtails overall fever duration (Wu et al., 2023, 2024). Furthermore, combining electronic fibrobronchoscope alveolar lavage with the local administration of inhaled terbutaline, budesonide, or acetylcysteine instillation directly at the lesion site drastically lowers local inflammatory factors, minimizes procedural trauma, and significantly reduces the risk of long-term sequelae like bronchiolitis obliterans (Sheng et al., 2024; Xu et al., 2024b; Li et al., 2025b).

## 6 Bridging the translational gap: next-generation experimental models

A major bottleneck in unraveling the intricate pathogen-host dynamics of MPP lies in the limitations of traditional experimental models. Conventional 2D immortalized epithelial cell lines (e.g., A549) lack mucociliary differentiation, while murine models often fail to accurately recapitulate the complex, pseudostratified architecture and species-specific immune receptor profiles of the human respiratory tract (Humbert et al., 2022).

To truly decode how *M. pneumoniae* subverts host immunity, recent research has successfully pivoted toward advanced, high-throughput *in vitro* platforms (Mahieu et al., 2024). A prominent example is the use of primary human bronchial epithelial cells (hBECs) cultured at Air-Liquid Interface (ALI). These ALI models successfully differentiate into basal, ciliated, and goblet cells, faithfully recreating physiological mucociliary clearance and pathogen-induced mucus hypersecretion (Hao et al., 2014). This sophisticated architecture has allowed researchers to directly visualize the spatial and temporal colonization patterns of *M. pneumoniae*, demonstrating how the bacteria utilize gliding motility to initially bind to ciliated cells and subsequently establish persistent infections (Prince et al., 2014).

Crucially, these advanced platforms are instrumental in modeling chronic infections and addressing the escalating antimicrobial resistance (AMR) crisis, characterized by widespread MRMP and biofilm-associated immune evasion (Rowlands et al., 2024). Unlike simple 2D cultures, ALI systems produce human-like mucus layers where *M. pneumoniae* can form robust “biofilm towers” associated with ciliary tips, a phenotype that provokes a tempered inflammatory response and promotes chronic persistence (Fahim et al., 2026). This physiological representation allows researchers to accurately test the efficacy of novel therapeutics under genuine infection conditions. For instance, it provides a reliable platform to evaluate biofilm-disrupting agents in combination with alternative antibiotics, such as doxycycline, which has demonstrated crucial clinical efficacy against MRMP outbreaks (Douvoyiannis et al., 2026; Gross et al., 2010). Furthermore, these platforms serve as a vital preclinical bridge for HDTs, enabling the simultaneous assessment of a drug’s capacity to suppress epithelial inflammatory cascades and its potential cytotoxic effects prior to initiating clinical trials (Humbert et al., 2022).

## 7 Conclusion and future perspectives

The post-pandemic landscape of pediatric Mycoplasma pneumoniae pneumonia has mandated a paradigm shift from a purely pathogen-centric perspective to a holistic host-pathogen interaction framework. The dramatic rise of SMPP, RMPP, and MRMP is a manifestation of profound immune dysregulation, encompassing T-cell exhaustion, inflammasome-mediated pyroptosis, extensive endothelial breakdown, and disruption of the gut-lung axis.

By harnessing multi-omics phenotyping, clinicians can now translate biological noise into actionable host-response biomarker signatures, utilizing nomograms and machine learning for early risk stratification. Looking forward, the integration of Artificial Intelligence (AI)-driven Clinical Decision Support Systems (CDSS) with targeted next-generation sequencing (tNGS) and the deployment of 3D airway organoid models will pave the way for personalized Host-Directed Therapies. Exploring targeted pathway inhibitors (e.g., JAK inhibitors), precise anticoagulation, and microbiota-modulating interventions holds the greatest promise for mitigating severe lung injury, preventing catastrophic complications, and securing optimal long-term respiratory health for children worldwide.

However, current advancements must be interpreted with caution regarding geographical bias. The majority of robust multi-omics data, machine-learning nomograms, and epidemiological surveillance of the A2063G MRMP surge are derived from East Asian cohorts. Future endeavors must prioritize global, multicenter prospective cohorts to validate these biomarker signatures across diverse ethnic populations and healthcare settings.
